# Flavonifractor porci sp. nov. and Flintibacter porci sp. nov., two novel butyrate-producing bacteria of the family Oscillospiraceae

**DOI:** 10.1099/ijsem.0.006767

**Published:** 2025-04-30

**Authors:** Han-Yu Niu, Jie Zhang, Hao-Jie Huang, Xin-Wei Sun, Hao-Yu Chen, Xiao-Meng Wang, Ci Liu, Ming-Xia Bi, Shuang-Jiang Liu

**Affiliations:** 1 College of Veterinary Medicine, Shanxi Agricultural University (Shanxi Academy of Agricultural Sciences), Jinzhong, 030801, PR China; 2 State Key Laboratory of Microbial Technology, Shandong University, Qingdao, 266237, PR China; 3 State Key Laboratory of Microbial Resources and Environmental Research Center, Institute of Microbiology, Chinese Academy of Sciences, Beijing, 100101, PR China

**Keywords:** *Flavonifractor porci *strain P01024^T^, *Flintibacter porci *strain P01025^T^, *Oscillospiraceae*, pig gut microbiome, polyphasic taxonomy

## Abstract

Two Gram-stain-negative, strictly anaerobic, non-motile, non-­spore-forming and rod-shaped bacterial strains, namely, P01024^T^ and P01025^T^, were isolated from piglet manure. The strains P01024^T^ and P01025^T^ fermented glucose to acetate and butyrate. The major cellular fatty acids (>10.0%) of strain P01024^T^ were C_14 : 0_, C_16 : 0_ and summed feature 9 (iso-C_17:1_ *ω*9c and/or 10-Methyl-C_16:0_) and of strain P01025^T^ were C_14 : 0_, C_16 : 0_ and summed feature 3 (C_16 : 1_* ω6*c and/or C_16 : 1_* ω7*c). Analysis of 16S rRNA gene sequences indicated that strains P01024^T^ and P01025^T^ belonged to the family *Oscillospiraceae*. The strain P01024^T^ showed high identities of 16S rRNA genes to type species *Flavonifractor plautii* ATCC 29863^T^ (96.64%). The highest percentages of conserved protein (POCP) value between strain P01024^T^ and *Flavonifractor plautii* ATCC 29863^T^ was 59.84%. The average nt identity (ANI) and digital DNA–DNA hybridization (dDDH) values between strain P01024^T^ and *Flavonifractor plautii* ATCC 29863^T^ were 79.51% and 23.80%, respectively, supporting that strain P01024^T^ represented a novel species of the genus *Flavonifractor*. Strain P01025^T^ showed high identities of 16S rRNA genes to the type species *Flintibacter butyricus* BLS21^T^ (95.87%). The highest POCP and AAI (average aa identity) values of strains P01025^T^ to *Flintibacter hominis* New-19^T^ were 53.02% and 73.11%, respectively. The ANI and dDDH values between strains P01025^T^ and *Flintibacter hominis* New-19^T^ were 75.44% and 23.40%, respectively, supporting that strain P01025^T^ represented a novel species in the genus *Flintibacter*. The calculated G+C molar contents for strains P01024^T^ and P01025^T^ were 58.43 and 56.44 mol%, respectively. Together with phenotypic features, we concluded that strains P01024^T^ and P01025^T^ represented novel species in the genera *Flavonifractor* and *Flintibacter* of the family *Oscillospiraceae*, respectively, for which the names *Flavonifractor porci* sp. nov. (type strain P01024^T^=CGMCC 1.18055^T^=KCTC 25793^T^) and *Flintibacter porci* sp. nov. (type strain P01025^T^=CGMCC 1.18060^T^=KCTC 25794^T^) are proposed.

## Introduction

Pigs represent a livestock species of significant economic value and provide the world’s most consumed meat [1,2]. Pigs possess monogastric digestive systems and serve as suitable animal models for biomedical studies [3,5]. Studies reported that gut microbiomes and their metabolites such as short-chain fatty acids (SCFAs) were associated with host health and affected the development and growth of piglets [6]. Metagenomic analysis of pig gut microbiome indicates that 84.20% of metagenome-assembled genomes were not able to be classified into any known taxon [7]. These highlight the importance and necessity of isolation, cultivation and characterization of pig gut microbes.

The family *Oscillospiraceae* is an important component of the gut microbiome. Previous studies reported that the members of *Oscillospiraceae* contained taxa specialized in the decomposition of complex plant materials and the production of SCFAs [8]. At the date of writing, the family *Oscillospiraceae* comprises 64 genera with validly published names (https://lpsn.dsmz.de/family/oscillospiraceae). The genera *Flavonifractor* [9] and *Flintibacter* [10] are members of the family *Oscillospiraceae*, and their type species were isolated from manure samples. The genus *Flavonifractor* has eight species, one of which has a validly published name, while the remaining seven are designated under the provisional Candidatus status (https://lpsn.dsmz.de/genus/flavonifractor), and the genus *Flintibacter* has four species, three of which are assigned validly published names (https://lpsn.dsmz.de/genus/flintibacter).

In this study, we isolated and characterized two strains P01024^T^ and P01025^T^ of the family *Oscillospiraceae*, from piglet manure. Based on the results of polyphasic studies, strains P01024^T^ and P01025^T^ were identified as novel species of the genus *Flavonifractor* and genus *Flintibacter* from *Oscillospiraceae*, respectively, for which the names *Flavonifractor porci* and *Flintibacter porci* are proposed.

## Methods

### Animal manure sampling

Manure samples were collected from two healthy piglets, aged 21–28 days, located at Qinhuangdao, Hebei, China. All manure samples were collected non-invasively from piglets in a commercial swine farm under natural conditions without any human-induced physiological or behavioural interference. After collection, the samples were immediately frozen in liquid nitrogen, placed in anaerobic bags with deoxygenation packs and shipped on dry ice. All samples were stored at −80 °C and used for bacterial cultivation.

### Culture media, bacterial isolation, cultivation and preservation

The medium used in this experiment was modified mGAM (mmGAM) medium, which was based on the modified GAM (mGAM) (HB8518, Hopebio, China) with the following additional components (per litre of distilled water): 0.5 g l-cysteine hydrochloride (C14772120, Macklin, China), 0.5 g l-arginine (H2113165, Aladdin, China), 0.3 g l-tryptophan (C11677452, Macklin), 2.0 g NaHCO_3_ (10018960, Sinopharm Group, China), 2.46 g CH_3_COONa (C15676262, Macklin), 5 ml haemoglobin chloride (C11677452, Macklin), 1 ml resazurin (C11677452, Macklin), 100 ml clarified rumen fluid (A110101, Wizbiotech, China), 50 ml goat blood (HQ60071, Hongquan Bio, China), 5 ml vitamin K1 solution (HB8462, Hopebio), 1 ml Wolfe’s vitamin solution (SL0110, Coolaber, China) and 1 ml Wolfe’s mineral solution (SL0120, Coolaber). The d-mannose (C14246372, Macklin), d-fructose (C12015855, Macklin), palatinose hydrate (E2025150, Aladdin), inulin (C15492472, Macklin), d-galactose (C15131463, Macklin), palatinose (C14786871, Macklin), l-rhamnose (C15884351, Macklin), cellobiose (B2311508, Aladdin) and trehalose (C14894221, Macklin) were 0.5 g l^−1^. The pH was adjusted to 7.2 [11].

The stored manure samples were thawed gradually on ice. All operations were conducted in an anaerobic workstation (AW 500SG, Electrotek) filled with 85% N_2_, 10% H_2_ and 5% CO_2_. One gramme of the manure sample was suspended in 9 ml PBS buffer (P1020, Solarbio, China) and filtered through a cell strainer (15-1040, Biologix, China) with a pore size of 40 µm. The filtrate was serially diluted (up to 10^−7^) with PBS buffer, and the parts of 10^−4^ to 10^−7^ dilutions were spread into mmGAM agar plates. Inoculated agar plates were anaerobically incubated at 37 °C for 3–10 days. Strain P01024^T^ was isolated on day 4 of incubation, and strain P01025^T^ was isolated on day 8. Bacterial colonies were picked up and inoculated into mmGAM liquid medium and subsequently incubated at 37 °C. Bacterial strains were preserved as previously described [11]. Strains P01024^T^ and P01025^T^ were stored at −80 °C in the mmGAM medium supplemented with 20% (v/v) glycerol and were deposited at the China General Microbiological Culture Collection Centre (CGMCC) and the Korean Collection for Type Cultures (KCTC) under the accessions of CGMCC 1.18055^T^=KCTC 25793^T^ and CGMCC 1.18060^T^=KCTC 25794^T^, respectively.

### Morphology, physiology and chemotaxonomy

Single colonies were cultured on mmGAM agar plates for 3 days at 37 °C. Gram staining (G1060, Solarbio) and spore staining (G1132, Solarbio) were performed, and the results of spore formation and Gram staining were observed with optical microscopy (Eclipse Ts2R, Nikon, Japan). Colony morphology was observed by stereoscopic microscope (DS-F2.5, Nikon). Cellular morphology was studied using transmission electron microscopy (JEM-1400, Jeol, Japan). Cell motility was assessed using mmGAM medium supplemented with 0.5% agar. Tolerance to molecular O_2_ was determined in mGAM medium without cysteine addition.

To determine the effect of temperature and pH on growth, the bacterial strains were cultivated at different temperatures (20, 25, 30, 35, 40, 45, 50 and 55 °C), or at different pH values (pH 4.0, 5.0, 5.5, 6.0, 6.5, 7.0, 7.5, 8.0, 9.0 and 10.0) at 37 °C. Sterile solutions of CH_3_COOH-CH_3_COONa buffer (pH=3.6) were used to adjust acidity (pH 4.0–5.0), and Na_2_HPO_4_/NaH_2_PO_4_ buffer (pH 6.0–8.0) and NA_2_CO_3_-NAHCO_3_ buffer (pH=10.3) were used to adjust alkalinity (pH 9.0–10.0). The growth was measured with a UV/visible spectrophotometer at the wavelength of 600 nm (OD_600_) (Harvard Biochrom Ultrospec 10, UK), and all experiments were conducted in triplicate.

Susceptibility to antibiotics was tested using the disc diffusion method on Mueller–Hinton agar (Bikeman, China, microgramme per disc unless otherwise stated) according to the Eucast standards [12]. Antibiotic susceptibility of strains P01024^T^ and P01025^T^ was determined by measuring the diameter of the inhibitory zone, including (per disc) 15 µg erythromycin, 5 µg rifampin, 5 µg cefixime, 15 µg clarithromycin, 30 µg vancomycin, 30 µg tetracycline, 10 µg gentamicin, 15 µg azithromycin, 100 µg carbenicillin, 10 µg streptomycin, 300 IU polymyxin, 30 µg kanamycin, 20 µg clindamycin, 10 µg penicillin, 30 µg cefoperazone, 5 µg ciprofloxacin, 10 µg amoxicillin, 0.04 µg bacitracin, 30 µg chloromycetin or 10 µg ampicillin. Inhibitory zone diameters were then measured after anaerobic culture at 37 °C for 2 days [13]. All tests were performed in triplicate.

Physiological and biochemical traits were determined by using the API 20A Anaerobe Test Kit (bioMérieux, France), AN MicroPlates (1007, BIOLOGY, USA) [14] and the API ZYM Kit (bioMérieux), according to the manufacturer’s instructions. To determine SCFAs, the strains P01024^T^ and P01025^T^ were cultured in mmGAM broth for 2 days. Fresh bacterial cultures were mixed with ethyl acetate, and after repeating centrifugation and standing steps, the supernatant was taken for GC-MS analysis. The sample was analysed using a GC-MS-QP2010 Ultra instrument equipped with an autosampler and a DB-wax capillary column (30 m long, 0.25 mm inner diameter and 0.25 µm film thickness, Shimadzu) [15]. The whole-cell fatty acids and polar lipids were determined according to the methods reported previously [15,17].

### 16S rRNA gene phylogeny

The 16S rRNA gene sequences were amplified using universal primers 27F (5′-GAGAGTTTGATCCTGGCTCAG-3′) and 1492R (5′-TACGGYTACCTTGTTACGACTT-3′) [18]. DNA sequencing was performed by Beijing Tsingke Biotech Co., Ltd. (Qingdao, China) using PCR products. The 16S rRNA gene sequences of strains P01024^T^ and P01025^T^ were blasted with the available sequences in the databases NCBI (https://www.ncbi.nlm.nih.gov/) and EzBioCloud (https://www.ezbiocloud.net/) [19]. The 16S rRNA gene sequences of 64 type strains in the family *Oscillospiraceae* and all strains in the genus *Pseudoflavonifractor*, *Flavonifractor*, *Flintibacter* and *Muriventricola* were downloaded from EzBioCloud and NCBI. Phylogenetic analysis based on 16S rRNA gene sequences was carried out using mega-X [20] with the neighbour-joining (NJ), maximum-likelihood (ML) and maximum-parsimony (MP) methods [15,21,23]. The Kimura two-parameter model was used to reconstruct phylogenetic trees [18]. Branching patterns of the NJ tree were evaluated by bootstrapping with 1,000 replicates [24].

### Genome features

The genomic DNA of the strains was extracted using the TIANamp Bacteria DNA Kit (DP302, Tiangen, China) following the instruction of the manufacturer. Genome sequencing was performed using the Illumina and PacBio platforms at Guangdong MAGiGENE Biotechnology company (https://www.magigen.com). The genomic assembly was performed with the SPAdes software (version 3.9.0) [25]. The genome sequence was deposited in the NCBI GenBank database and assigned the accession number. To evaluate the phylogenomic assignment of the novel species, the average nt identity (ANI) and digital DNA–DNA hybridization (dDDH) values, percentages of conserved protein (POCP), the percentage of average aa identity (AAI) and its phylogenetic neighbours were calculated; ANI was calculated by using the web-based ANI calculator EzBioCloud, and an unweighted arithmetic mean dendrogram (UPGMA) was generated using the Orthologous Average Nucleotide Identity Tool (OAT) version 0.93.1 [26]. The dDDH values were calculated by Genome-to-Genome Distance Calculator (GGDC) 3.0 online service [27]. The POCP was calculated according to a previously reported method [28]. The AAI values were calculated using EzAAI [29]. The phylogenetic tree based on the whole genome was constructed using the CVTree method [30,31]. In addition, the obtained genome sequences were annotated using the Kyoto Encyclopedia of Genes and Genomes (KEGG) databases [32].

## Results

### Morphology, physiology and growth characteristics

**Fig. 1.**
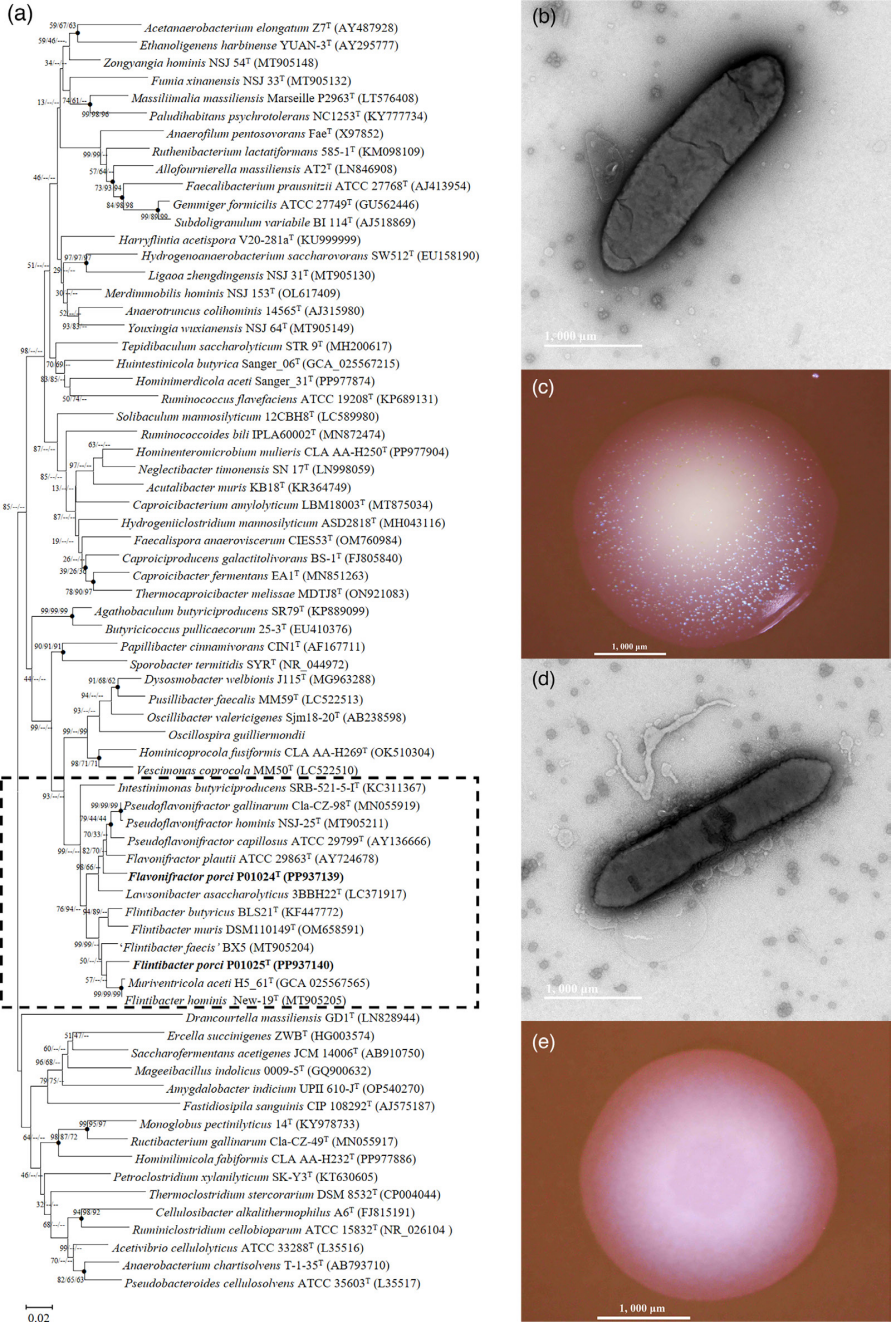
The phylogeny and morphology of strains P01024^T^ and P01025^T^. (**a**) The phylogenetic tree was constructed using the NJ algorithm with 16S rRNA genes of P01024^T^, P01025^T^ and all type species of the family *Oscillospiraceae*. The new names proposed in this study are shown in bold. The 16S rRNA gene sequences of type species were downloaded from GenBank, and accession numbers are given in parentheses. Filled circles indicate branches that were also found in trees generated with the ML and MP algorithms. Numbers at branch nodes represent the percentage of associated taxa clustered together in the bootstrap test (1,000 replicates) (NJ/ML/MP). Bar, 0.02 substitutions per nt position. (**b, d**) Transmission electron microscope images of cells. (**c, e**) Stereoscopic microscope images of colonies.

During extensive efforts to harvest microbial diversity, we cultivated bacteria from pig manure samples, and the strains, namely, P01024^T^ and P01025^T^, were obtained. Phylogenetic analysis according to 16S rRNA gene suggested that P01024^T^ and P01025^T^ were closely related to type species of the family *Oscillospiraceae* and were possibly two novel species (Fig. 1a). Strain P01024^T^ was non-motile, Gram-negative, non-spore forming and rod shaped (3.5–4 µm long×0.8–1 µm wide) (Fig. 1b). Colonies after anaerobic incubation on mmGAM agar plates for 3–5 days were 3–4 mm in diameter, translucent whitish, circular, convex, slightly opaque and sparkling (Fig. 1c). Growth occurred at temperatures ranging from 30 to 55 °C (optimum temperature 37 °C), and the pH range for growth was 5.5–9.0 (optimum pH 7.5). No growth was observed in the mmGAM liquid medium containing 0.3% of ox gall powder, or in the presence of oxygen in the mmGAM liquid medium at 37 °C for 3 days, indicating that strain P01024^T^ was a strictly anaerobic bacterium.

The strain P01025^T^ was non-motile, Gram-negative, non-spore forming and rod shaped (3.2–4 µm long×0.2–0.3 µm wide). The results from transmission electron microscopy showed that strain P01025^T^ secreted large numbers of exosomes at the late logarithmic phase of growth (Fig. 1d). Colonies after anaerobic incubation on mmGAM agar plates for 3–5 days were 2–3 mm in diameter, translucent whitish, circular and convex (Fig. 1e). Growth occurred at temperatures ranging from 25 to 45 °C (optimum temperature 37 °C), and the pH range for growth was 5.5–9.0 (optimum pH 7). No growth was observed in the mmGAM liquid medium containing 0.3% ox gall powder, or in the presence of oxygen incubating at 37 °C for 3 days in the mmGAM medium, indicating that strain P01025^T^ was strictly anaerobic.

### Phenotypic characteristics of strains P01024^T^ and P01025^T^

Strain P01024^T^ was sensitive to chloramphenicol, penicillin, carbenicillin, ampicillin, clindamycin, amoxicillin, cefoperazone, tetracycline, azithromycin, vancomycin, clarithromycin and erythromycin. In the API ZYM strip, there were positive results for alkaline phosphatase, esterase (C4), esterase lipase (C8), leucine arylamidase, acid phosphatase and naphthol-AS-BI-phosphohydrolase and negative results for lipase (C14), valine arylamidase, cystine arylamidase, trypsin, *α*-chymotrypsin, *α*-galactosidase, *β*-galactosidase, *β*-glucuronidase, *α*-glucosidase, *β*-glucosidase, *N*-acetyl-*β*-glucosaminidase, *α*-mannosidase and *α*-fucosidase. The results of the API 20A test showed that strain P01024^T^ was positive for aescine iron (III) citrate *β*-glucosaccharase, but other indicators of assimilation were negative in the API 20A test strip. Biolog AN MicroPlates showed that cells metabolized *N*-acetyl-d-glucosamine, adonitol, amygdalin, d-arabitol, d-cellobiose, dextrin, i-erythritol, d-fructose, l-fucose, d-galactose, d-galacturonic acid, gentiobiose, d-glucosaminic acid, *α*-d-glucose, glucose-1-phosphate, glucose-6-phosphate, glycerol, *α*-d-lactose, lactulose, maltose, maltotriose, d-mannose, d-melezitose, d-melibiose, 3-methyl-d-glucose, *β*-methyl-d-galactoside, palatinose, d-raffinose, l-rhamnose, d-sorbitol, sucrose, d-trehalose, turanose, acetic acid, formic acid, fumaric acid, glyoxylic acid, *β*-hydroxybutyric acid, *α*-ketobutyric acid, *α*-ketovaleric acid, propionic acid, pyruvic acid, pyruvic acid methyl ester, l-serine, 2′-deoxy adenosine, inosine, thymidine, uridine, thymidine-5′-monophosphate, uridine-5′-monophosphate, weak metabolized *N*-acetyl-*β*-d-mannosamine, *α*-cyclodextrin, m-inositol, stachyose, *α*-hydroxybutyric acid, succinic acid, l-alanyl-l-threonine and l-threonine. The distinctive biochemical characteristics of strain P01024^T^ from its phylogenetically close neighbours are summarized in Table 1. All biochemical tests were performed in triplicate. The major products of strain P01024^T^ from glucose fermentation were acetate (5.84×10⁻³ g l⁻¹) and butyrate (0.124 g l⁻¹).

**Table 1.** Differential characteristics of strain P01024^T^ and the closely related species of the family *Oscillospiracea.*Notes: +, positive; −, negative; nd, no data available; v, variable; Summed feature 9* contains an iso-C_17 : 1_* ω9*c (15-methylhexadec-8-enoic acid) and/or 10-Methyl-C_16:0_ (10-Methylhexadecanoic acid)

	*Flavonifractor porci*P01024^T^	*Pseudoflavonifractor capillosus* ATCC 29799^T^ [9]	*Flavonifractor plautii* ATCC 29863^T^ [9]	*Pseudoflavonifractor hominis* NSJ-25^T^ [28]	*Pseudoflavonifractor gallinarum* Cla-CZ-98^T^ [38]
**Characteristic**					
Isolation source	Pig manure	Human faeces	Human faeces	Human faeces	Chicken caecum
Motility	−	−	v	−	nd
Gram stain	−	−	v	nd	nd
Spore formation	−	−	v	nd	nd
Temperature range (°C)	30–55	37–45	37	37	37
pH range	5.5–9.0	nd	nd	7.0–7.5	nd
**Enzyme activity**					
Alkaline phosphatase	+	nd	nd	nd	nd
Esterase (C4)	+	nd	nd	nd	nd
Esterase lipase (C8)	+	nd	nd	nd	nd
Leucine arylamidase	+	nd	nd	nd	nd
Acid phosphatase	+	nd	nd	nd	nd
Naphthol-AS-BI-phosphohydrolase	+	nd	nd	nd	nd
*β*-Galactosidase	−	nd	nd	nd	nd
*β*-Glucosidase	−	nd	nd	nd	nd
Aesculin hydrolysis	+	+	−	nd	nd
**DNA G+C content (mol%**)	58.43	60	58–61.6	49.22	59.9
**Major cellular fatty acids (>5% of totals**)	C_14 : 0_, C_16 : 0_, summed feature 9*	C_14 : 0_, C_16 : 0_	C_14 : 0_, C_16 : 0_	nd	C_14 : 0_, C_16 : 0_ DMA, C_14 : 0_ DMA, C_12 : 0_, C_18 : 0_ DMA
**Polar lipids**	PGP, PGL, PL, GL, L	nd	nd	nd	nd
**Metabolic end products**	Acetate, butyrate	Acetate, succinate	Acetate, butyrate	nd	nd

Strain P01025^T^ was sensitive to chloramphenicol, penicillin, carbenicillin, ampicillin, amoxicillin, cefoperazone, bacitracin and vancomycin. In the API ZYM strip, there were positive results for alkaline phosphatase, esterase (C4), esterase lipase (C8), acid phosphatase, naphthol-AS-BI-phosphohydrolase, *β*-galactosidase and *β*-glucosidase and negative results for lipase (C14), leucine arylamidase, valine arylamidase, cystine arylamidase, trypsin, *α*-chymotrypsin, *α*-galactosidase, *β*-glucuronidase, *α*-glucosidase, *N*-acetyl-*β*-glucosaminidase, *α*-mannosidase and *α*-fucosidase. Cells hydrolyse aesculin but not gelatin. Cells are negative for indole formation, catalase and urease based on the API 20A system, and other indicators of assimilation were negative in the API 20A test strip. Biolog AN MicroPlates showed that cells metabolized *N*-acetyl-d-glucosamine, *N*-acetyl-*β*-d-mannosamine, adonitol, d-fructose, l-fucose, d-galactose, d-galacturonic acid, gentiobiose, *α*-d-glucose, glucose-6-phosphate, d-mannose, d-melibiose, 3-methyl-d-glucose, palatinose, l-rhamnose, glyoxylic acid, *α*-ketobutyric acid, *α*-ketovaleric acid, l-malic acid, pyruvic acid, pyruvic acid methyl ester, l-serine, l-threonine, inosine, uridine, weak metabolized arbutin, d-cellobiose, dextrin, d-glucosaminic acid, *α*-d-lactose, lactulose, maltotriose, *β*-methyl-d-galactoside, turanose, l-alanine and l-glutamic acid. The distinctive biochemical characteristics of strain P01025^T^ from its phylogenetically close neighbours are summarized in Table 2. All biochemical tests were performed in triplicate. The major products of strain P01025^T^ from glucose fermentation were identified to be acetate (2.70×10⁻³ g l⁻¹) and butyrate (0.0933 g l⁻¹).

**Table 2.** Differential characteristics of strain P01025^T^ and the closely related species of the family *Oscillospiraceae.*Notes: +, positive; −, negative; nd, no data available; summed feature 3* contains a C_16 : 1_* ω6*c (*cis*-10-hexadecenoic acid) and/or C_16 : 1_* ω7*c (*cis*-9-hexadecenoic acid).

	*Flintibacter porci*P01025^T^	*Flintibacter butyricus* BLS21^T^ [10]	*Flintibacter muris* DSM110149^T^ [39]	*Flintibacter hominis* New-19^T^ [28]	*Muriventricola aceti* H5_61^T^ [40]
**Characteristic**					
Isolation source	Pig manure	Mouse manure	Mouse caecum	Human faeces	Human faeces
Motility	−	−	nd	−	nd
Gram stain	−	−	nd	nd	nd
Spore formation	−	−	nd	nd	nd
Temperature range (°C)	25–45	30–40	nd	37	nd
pH range	55–9.0	nd	nd	7.0–7.5	nd
**Enzyme activity**					
Alkaline phosphatase	+	nd	nd	nd	nd
Esterase (C4)	+	nd	nd	nd	nd
Esterase lipase (C8)	+	nd	nd	nd	nd
Leucine arylamidase	−	nd	nd	nd	nd
Acid phosphatase	+	nd	nd	nd	nd
Naphthol-AS-BI-phosphohydrolase	+	nd	nd	nd	nd
*β*-Galactosidase	+	nd	nd	nd	nd
*β*-Glucosidase	+	nd	nd	nd	nd
Aesculin hydrolysis	+	nd	nd	nd	nd
**DNA G+C content (mol%**)	56.44	58	55.8	60.49	55.8–56.3
**Major cellular fatty acids (>5% of totals**)	C_14 : 0_, C_16 : 0_, summed feature 3*	C_12 : 0_, C_14 : 0_, C_16 : 0_, C_18 : 0_, iso C_17 : 1_/anteiso C_17 : 1_, iso-C_19:1_, C_18 : 1_* ω9*c	C_18 : 0_ DMA, C_18 : 0_ FAME, C_14 : 0_ FAME, C_16 : 0_ FAME, C_16 : 0_ DMA, C_18 : 1_ cis9 DMA	nd	nd
**Polar lipids**	DPG, PGL, PL, GL, L	nd	nd	nd	nd
**Metabolic end products**	Acetate, butyrate	Acetate, butyrate	Acetate, carbon dioxide	nd	nd

We cultivated strains P01024^T^ and P01025^T^ in mmGAM broth and subjected to cellular fatty acid profiling in parallel in this study. The predominant cellular fatty acids (>10%) of strain P01024^T^ were C_14 : 0_ (19.6%), C_16 : 0_ (47.8%) and summed feature 9 (iso-C_17:1_ *ω*9c and/or 10-Methyl-C_16:0_) (29.7%). More detailed cellular fatty acids are presented in Table S1a (available in the online Supplementary Material). The major polar lipid of P01024^T^ was phosphatidylglycerol phosphate (PGP) and phosphoglycolipid (PGL). Several unidentified polar lipids, including an unidentified phospholipid (PL), three unidentified glycolipids (GLs) and nine unidentified lipids (Ls), were also detected (Fig. S1a).

The predominant cellular fatty acids (>10%) of strain P01025^T^ were C_14 : 0_ (25.6%), C_16 : 0_ (40.8%) and summed feature 3 (C_16 : 1_* ω6*c and/or C_16 : 1_* ω7*c) (19.8%). More detailed cellular fatty acids are presented in Table S1b. The major polar lipid of P01025^T^ was diphosphatidylglycerol (DPG) and two PGLs. Several unidentified polar lipids were also detected, including two unidentified PLs, three unidentified GLs and four unidentified Ls (Fig. S1b).

### Genomic characteristics of strains P01024^T^ and P01025^T^

The genome size of strain P01024^T^ was 3,351,356 bp, of which 3,204 coding sequences, including 56 tRNA genes, 5 rRNA genes (containing one 16S rRNA gene) and 10 sRNA genes. The total length of these coding regions accounted for 88.73% of the genome. The genome analysed with KEGG indicated that the majority of the genes are related to aa metabolism (131), carbohydrate metabolism (128), metabolism of cofactors and vitamins (107), membrane transport (131), translation (84), nt metabolism (78), energy metabolism (60), replication and repair (50).

The size of the strain P01025^T^ genome was 3,361,386 bp, of which 3,270 coding sequences, including 56 tRNA genes and 5 rRNA genes (which contain one 16S rRNA gene) and 8 sRNA. The total length of these coding regions accounted for 87.48% of the genome. The genome analysed with KEGG indicated that the majority of the genes are related to aa metabolism (140), carbohydrate metabolism (135), membrane transport (103), metabolism of cofactors and vitamins (90), translation (81), nt metabolism (77), energy metabolism (74) and signal transduction (58).

### Phylogeny and taxonomy of strains P01024^T^ and P01025^T^

**Fig. 2.**
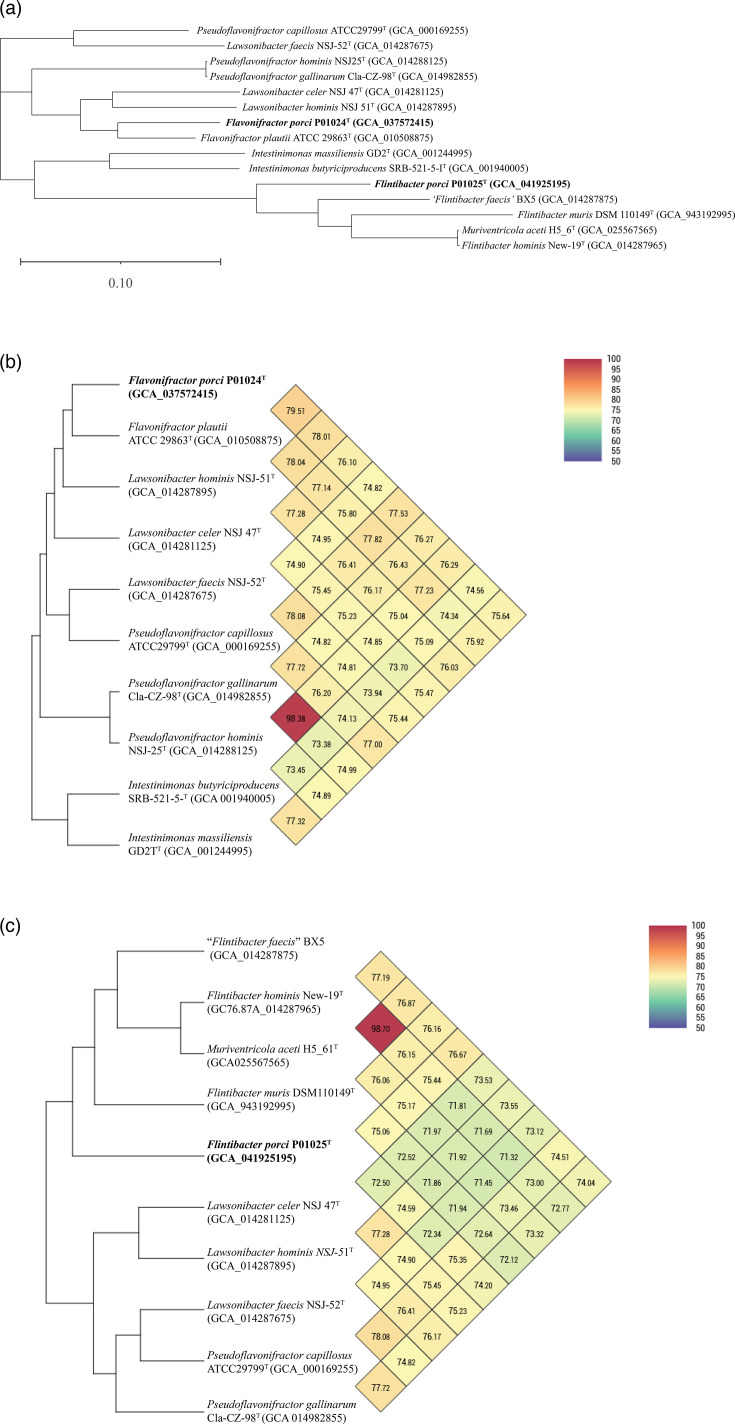
Phylogenomic and UPGMA phylogenetic trees and ANI heat maps of strains P01024^T^ and P01025^T^. (**a**) Phylogenomic trees of strains P01024^T^ and P01025^T^ and their phylogenetically closely related neighbours. Phylogenomic tree based on whole genomes was reconstructed using the CV Tree model. The new names proposed in this study are shown in bold. (**b**) The UPGMA phylogenetic trees and the ANI heat maps display the connections between strains P01024^T^ and their closely related neighbours. (**c**) The UPGMA phylogenetic trees and the ANI heat maps display the connections between strains P01025^T^ and their closely related neighbours. GenBank accession numbers are given in parentheses. Superscript ‘^T^’ or (T) designates type strain.

The identities of 16S rRNA genes between strain P01024^T^ and closely related type strains ranged from 86.83% to 97.1%. The low 16S rRNA gene identity values suggested that strain P01024^T^ possibly represented a novel species [33]. Analysis of 16S rRNA gene sequences indicated that strain P01024^T^ was phylogenetically close to members of genera *Flavonifractor*, *Lawsonibacter* and *Pseudoflavonifractor*, and it was difficult to assign it to any of the above genus. Thus, we further calculated the POCP and the AAI values between P01024^T^ and the type species of *Flavonifractor*, *Lawsonibacter* and *Pseudoflavonifractor*. Results showed that the POCP values of strain P01024^T^ to *Flavonifractor plautii* ATCC 29863^T^, *Lawsonibacter celer* NSJ 47^T^ and *Pseudoflavonifractor capillosus* ATCC 29799^T^ were 59.8%, 55.8%, 51.0%, respectively. The POCP value between strain P01024^T^ and *Flavonifractor plautii* ATCC 29863^T^ (59.8%) surpassed the 50% genus delineation threshold [34], further supporting its phylogenetic placement within the genus *Flavonifractor*. The POCP values between strain P01024^T^ and closely related type species are summarized in Table S2a. The AAI values of strain P01024^T^ to *Flavonifractor plautii* ATCC 29863^T^, *Lawsonibacter celer* NSJ 47^T^ and *P. capillosus* ATCC 29799^T^ were 77.2%, 73.3% and 72.2%, respectively, with the highest value (77.2%) exceeding the proposed genus-level threshold of 60%–65% [33]. The higher POCP (59.8%) and AAI (77.2%) values were observed between P01024^T^ and *Flavonifractor plautii* ATCC 29863^T^, supporting that P01024^T^ was a member of the genus *Flavonifractor*. The ANI values between strain P01024^T^ and the species of *Flavonifractor* (79.5%), *Lawsonibacter* (74.8%–78.0%) and *Pseudoflavonifractor* (76.3%–77.5%) were below the boundary of 95% for delineation of species [35], and the ANI-based UPGMA dendrogram of strain P01024^T^ is presented in Fig. 2(b). The POCP and ANI values supported that strain P01024^T^ is from a new species, but not a new genus. The dDDH values between strain P01024^T^ and *Flavonifractor plautii* ATCC 29863^T^, *Lawsonibacter faecis* NSJ-52^T^, *Lawsonibacter celer* NSJ 47^T^, *Lawsonibacter hominis* NSJ-51^T^, *Pseudoflavonifractor hominis* NSJ-25^T^, *Pseudoflavonifractor gallinarum* Cla-CZ-98^T^ and *P. capillosus* ATCC 29799^T^ were 23.8%, 21.3%, 21.4%, 23.6%, 22.5%, 24.0% and 23.5%, respectively. These values were much lower than the thresholds of the dDDH (60%–70%) for delineation of species [36]. The highest ANI value (79.5%) and dDDH value (23.80%) were observed between P01024^T^ and *Flavonifractor plautii* ATCC 29863^T^, indicating that strain P01024^T^ represented a novel species in the genus *Flavonifractor* and was most closely related to *Flavonifractor plautii* ATCC 29863^T^. The above results supported that P01024^T^ represented a novel species of the genus *Flavonifractor*. The phylogenetic and the phylogenomic trees of the genus *Flavonifractor*, based on the 16S rRNA gene sequences and genomes, are shown in Figs 1(a) and 2(a). Phylogenetic trees reconstructed using the ML and MP methods are provided in Fig. S2a and S2b.

The identities of 16S rRNA genes between strain P01025^T^ and closely related type strains ranged from 87.5% to 97.0%. The low 16S rRNA gene identity values suggested that strain P01025^T^ possibly represented a novel species. Phylogenetic analysis of 16S rRNA gene sequences indicated that strain P01025^T^ was close to members of genera *Flintibacter* and *Muriventricola* within the family *Oscillospiraceae*. The POCP values of strains P01025^T^ to *Flintibacter hominis* New-19^T^ and to *Muriventricola aceti* H5_61^T^ were 53.0% and 50.5%, respectively. The POCP value between strain P01025^T^ and *Flintibacter hominis* New-19^T^ (53.0%) surpassed the 50% genus delineation threshold [34], further supporting its phylogenetic placement within the genus *Flintibacter*. The POCP values between strain P01025^T^ and related type species of closely related genera are summarized in Table S2a. The AAI values of strains P01025^T^ to *Flintibacter hominis* New-19^T^ and *M. aceti* H5_61^T^ were 73.1% and 72.8%, respectively. The highest POCP value (53.0%) and AAI value (73.1%) were observed between P01025^T^ and *Flintibacter hominis* New-19^T^, indicating that P01025^T^ was a member of the genus *Flintibacter*. The ANI values between strains P01025^T^ and the species of genera *Flintibacter* were 75.1%–76.7%, which were below the boundary of 95% for delineation of species [35]. The POCP and ANI values supported that strain P01024^T^ is from a new species, but not a new genus. The top ANI value was observed between strains P01025^T^ and ‘*Flintibacter faecis*’ BX5 (76.7%) and secondly between P01025^T^ and *Flintibacter hominis* New-19^T^ (75.4%). Nevertheless, the name ‘*Flintibacter faecis*’ has not been validated according to the rules of the International Code of Nomenclature of Prokaryotes [37]. The ANI-based UPGMA dendrogram of strain P01025^T^ is presented in Fig. 2(c). The dDDH values of strain P01025^T^ to *Flintibacter hominis* New-19^T^ and *Flintibacter muris* DSM110149^T^ were 23.4% and 22.3%, indicating that strain P01025^T^ represented a novel species in the genus *Flintibacter*. Phylogenetic and the phylogenomic trees of strain P01025^T^ and the genus *Flintibacter* are shown in Figs 1(a) and 2(a). The above results support that P01025^T^ represented a novel species of the genus *Flintibacter*. Additionally, when comparing the strains *M. aceti* H5_61^T^ and *Flintibacter hominis* New-19^T^, we found that the POCP value was 86.5%, the AAI value was 98.6%, the dDDH value was 90.9% and the ANI value was 98.7%. These values were above the values recommended for species delineation, suggesting that the taxonomic assignment of *M. aceti* H5_61^T^ requires re-evaluation.

Based on the distinct phenotypic, phylogenetic and genomic characteristics, strains P01024^T^ and P01025^T^ were considered as novel species of the genera *Flavonifractor* and *Flintibacter*, respectively, within family *Oscillospiraceae*, for which the names *Flavonifractor porci* sp. nov. (type strain P01024^T^=CGMCC 1.18055^T^=KCTC 25793^T^) and *Flintibacter porci* sp. nov. (type strain P01025^T^=CGMCC 1.18060^T^=KCTC 25794^T^) are proposed. Detailed descriptions of two proposed novel species are presented below.

## Description of *Flavonifractor porci* sp. nov.

*Flavonifractor porci* (por’ci. L. gen. n. *porci*, of a pig)

Cells are non-motile, Gram-negative, non-spore forming and rod shaped (3.5–4 µm long×0.8–1 µm wide). On the mmGAM agar plates, colonies with a diameter of 3–4 mm are translucent whitish, circular, convex, slightly opaque and shiny when cultivated at 37 °C for 3–5 days. Growth occurs at temperatures ranging from 30 to 55 °C (optimum temperature 37 °C); the pH range for growth is from 5.5 to 9.0 (optimum pH 7.5) and cannot grow in an aerobic environment. The major products from glucose fermentation are acetate and butyrate. Positive for alkaline phosphatase, esterase (C4), esterase lipase (C8), leucine arylamidase, acid phosphatase and naphthol-AS-BI-phosphohydrolase. Cells hydrolyse aesculin but not gelatin, do not produce indole and have no urease or catalase activity. Substrates can be utilized of *N*-acetyl-d-glucosamine, adonitol, amygdalin, d-arabitol, d-cellobiose, dextrin, i-erythritol, d-fructose, l-fucose, d-galactose, d-galacturonic acid, gentiobiose, d-glucosaminic acid, *α*-d-glucose, glucose-1-phosphate, glucose-6-phosphate, glycerol, *α*-d-lactose, lactulose, maltose, maltotriose, d-mannose, d-melezitose, d-melibiose, 3-methyl-d-glucose, *β*-methyl-d-galactoside, palatinose, d-raffinose, l-rhamnose, d-sorbitol, sucrose, d-trehalose, turanose, acetic acid, formic acid, fumaric acid, glyoxylic acid, *β*-hydroxybutyric acid, *α*-ketobutyric acid, *α*-ketovaleric acid, propionic acid, pyruvic acid, pyruvic acid methyl ester, l-serine, 2′-deoxy adenosine, inosine, thymidine, uridine, thymidine-5′-monophosphate, uridine-5′-monophosphate, *N*-acetyl-*β*-d-mannosamine, *α*-cyclodextrin, m-inositol, stachyose, *α*-hydroxybutyric acid, succinic acid, l-alanyl-l-threonine and l-threonine. The major cellular fatty acids are C_14 : 0_, C_16 : 0_ and summed feature 9 (iso-C_17:1_ *ω*9c and/or 10-Methyl-C_16:0_). The major polar lipids are PGP, PGLs, an unidentified PL, three unidentified GLs and nine unidentified Ls.

The type strain, P01024^T^ (=CGMCC 1.18055^T^=KCTC 25793^T^), was isolated from the manure samples of piglets aged 21–28 days. The DNA G+C content of the type strain is 58.43 mol%. GenBank accession numbers for the whole genome and the 16S rRNA gene sequence of strain P01024^T^ are JBBHLB000000000 and PP937139, respectively.

## Description of *Flintibacter porci* sp. nov.

*Flintibacter porci* (por’ci. L. gen. n. *porci*, of a pig)

Cells are non-motile, Gram-negative, non-spore forming, anaerobic and rod shaped (3.2–4 µm long×0.2–0.3 µm wide). Colonies after anaerobic incubation on mmGAM agar plates for 3–5 days are 2–3 mm in diameter, translucent whitish, circular and convex. Growth occurs at temperatures ranging from 25 to 45 °C (optimum temperature 37 °C), and the pH range for growth is 5.5–9.0 (optimum pH 7). The strain P01025^T^ secretes numerous exosomes at the late logarithmic phase of growth. The major products from glucose fermentation are identified to be acetate and butyrate. Cells hydrolyse aesculin but not gelatin, do not use tryptophan to produce indole and have no urease or catalase activity. Positive for alkaline phosphatase, esterase (C4), esterase lipase (C8), acid phosphatase, naphthol-AS-BI-phosphohydrolase, *β*-galactosidase and *β*-glucosidase. Substrates can be utilized of *N*-acetyl-d-glucosamine, *N*-acetyl-*β*-d-mannosamine, adonitol, d-fructose, l-fucose, d-galactose, d-galacturonic acid, gentiobiose, *α*-d-glucose, glucose-6-phosphate, d-mannose, d-melibiose, 3-methyl-d-glucose, palatinose, l-rhamnose, glyoxylic acid, *α*-ketobutyric acid, *α*-ketovaleric acid, l-malic acid, pyruvic acid, pyruvic acid methyl ester, l-serine, l-threonine, inosine, uridine, arbutin, d-cellobiose, dextrin, d-glucosaminic acid, *α*-d-lactose, lactulose, maltotriose, *β*-methyl-d-galactoside, turanose, l-alanine and l-glutamic acid. The major cellular fatty acids are C_14 : 0_, C_16 : 0_ and summed feature 3 (C_16 : 1_* ω6*c and/or C_16 : 1_* ω7*c). The major polar lipids are DPG, two PGLs, two unidentified PLs, three unidentified GLs and four unidentified Ls.

The type strain, P01025^T^ (=CGMCC 1.18060^T^=KCTC 25794^T^), was isolated from the manure samples of piglets aged 21–28 days. The DNA G+C content of the type strain is 56.44 mol%. GenBank accession numbers for the whole genome and the 16S rRNA gene sequence of strain P01025^T^ are JBHEVB000000000 and PP937140, respectively.

## Supplementary material

10.1099/ijsem.0.006767 Uncited Supplementary Material 1.

## Abbreviations

AAI: average aa identity
ANI: average nt identity
dDDH: digital DNA–DNA hybridization
DPG: diphosphatidylglycerol
GLs: glycolipids
KEGG: Kyoto Encyclopedia of Genes and Genomes
Ls: lipids
mGAM: modified GAM
ML: maximum likelihood
mmGAM: modified mGAM
MP: maximum parsimony
NJ: neighbour joining
PGL: phosphoglycolipid
PGP: phosphatidylglycerol phosphate
PL: phospholipid
POCP: percentages of conserved protein
SCFAs: short-chain fatty acids
