# Supplementary material for: Flavonifractor porci sp. nov. and Flintibacter porci sp. nov., two novel butyrate-producing bacteria of the family Oscillospiraceae
Source: Int J Syst Evol Microbiol. 2025 Apr 30;75(4):006767. doi: 10.1099/ijsem.0.006767 (PMC12050419; doi:10.1099/ijsem.0.006767)
Supplement: Uncited Supplementary Material 1. [file ijsem-75-06767-s001.pdf]

Supplementary Figure S1a: Total lipid profile of strain P01024^{T} analyzed

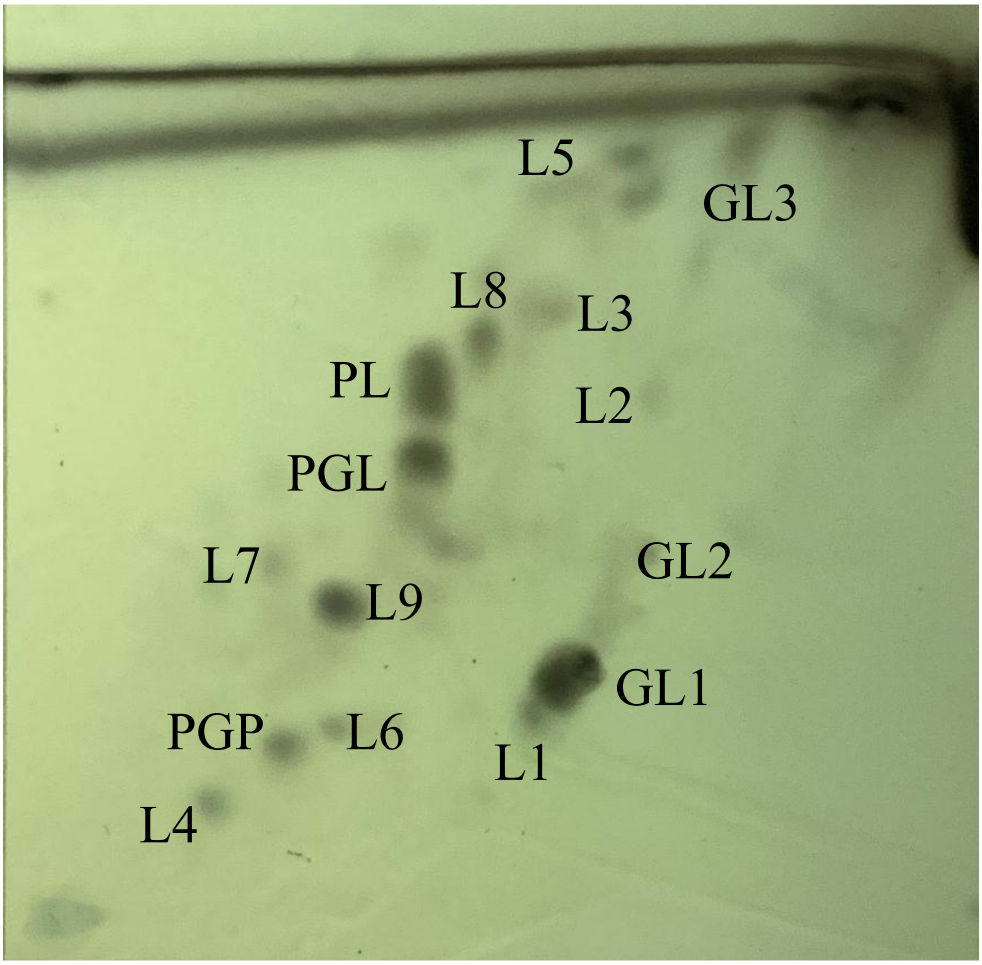

Abbreviations: PL, phospholipid; PGP, phosphatidylglycerol phosphate; PGL, phosphoglycolipid; GL, glycolipid; L, unidentified lipid.

Supplementary Figure S1b: Total lipid profile of strain P01025^{T} analyzed

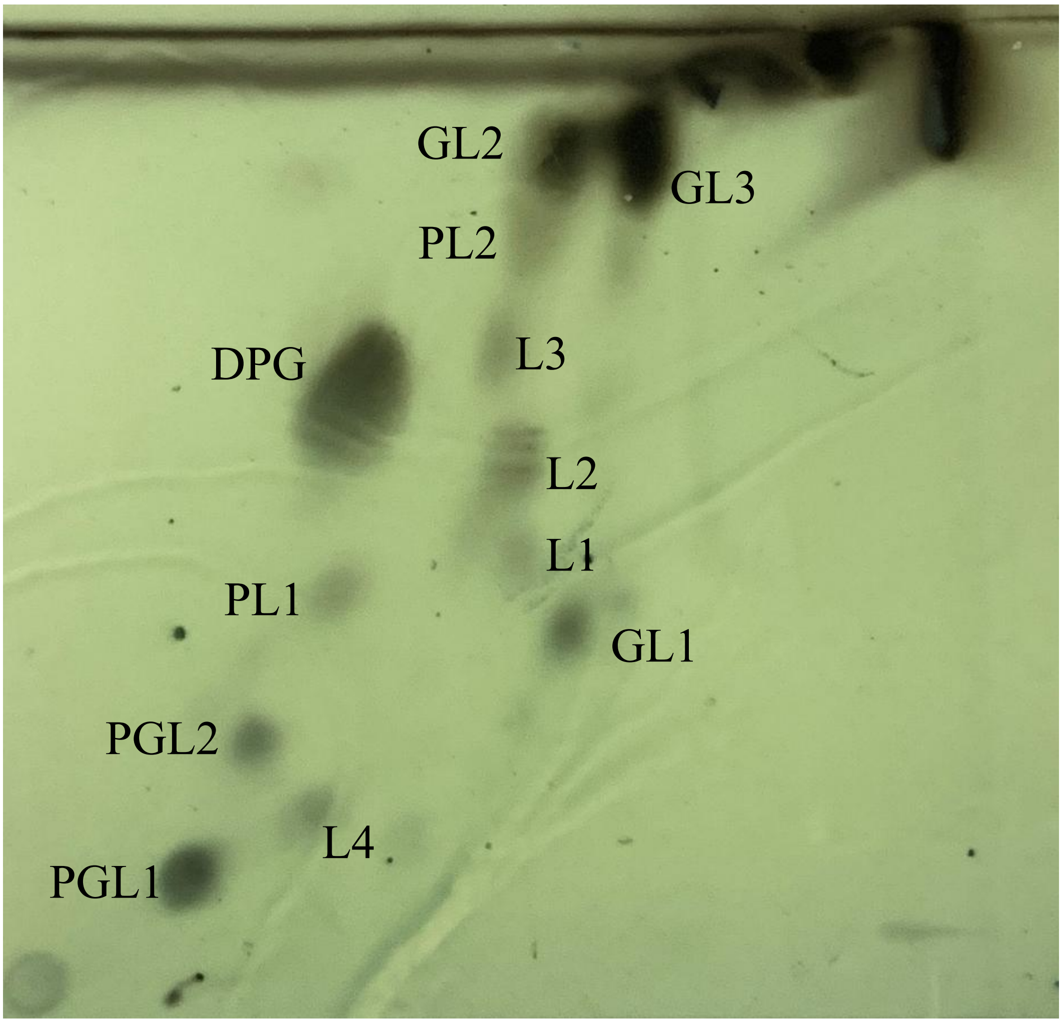

Abbreviations: PL, unidentified phospholipid; DPG, diphosphatidylglycerol; PGL, phosphoglycolipids; GL, unidentified glycolipid; L, unidentified lipid

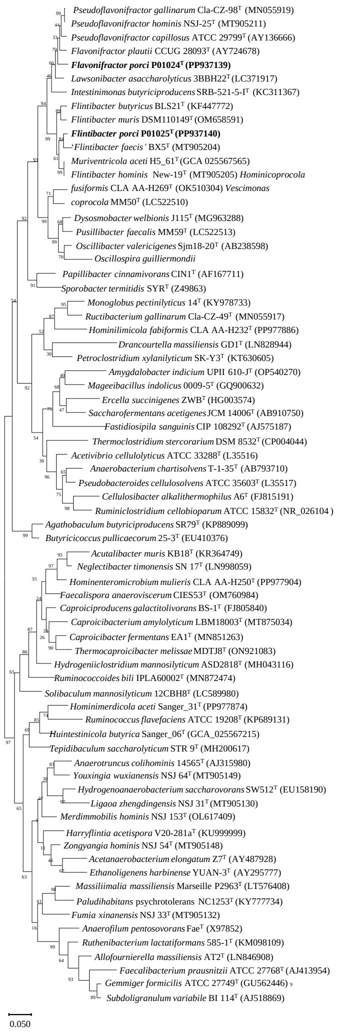

Supplementary Figure S2a: Maximum-Likelihood phylogenetic tree of strains P01024^{T} and P01025^{T}

The phylogenetic tree was constructed using Maximum-likelihood (ML) algorithm with 16S rRNA genes of P01024^{T}, P01025^{T} and all type species of the family *Oscillospiraceae*. The 16S rRNA genes sequences of type species were downloaded from GenBank, and accession numbers are given in parentheses. The new names proposed in this study are shown in bold.

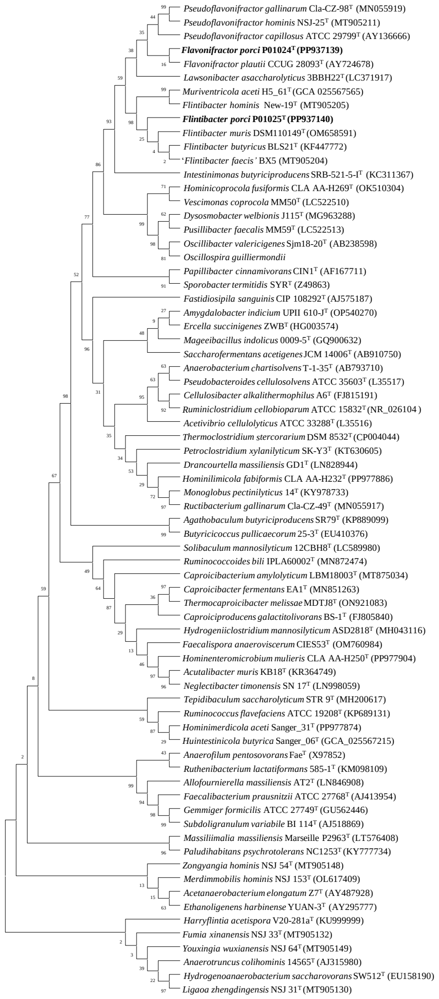

Supplementary Figure S2b: Maximum-Parsimony phylogenetic tree of strains P01024^{T} and P01025^{T}

The phylogenetic tree was constructed using Maximum-parsimony (MP) algorithm with 16S rRNA genes of P01024^{T}, P01025^{T} and all type species of the family *Oscillospiraceae*. The 16S rRNA genes sequences of type species were downloaded from GenBank, and accession numbers are given in parentheses. The new names proposed in this study are shown in bold.

RT	Response	Ar/Ht	RFact	ECL	Peak Name	Percent	Comment1	Comment2
0.7413	63119	0.006	----	6.5204		----	< min rt	
0.7512	1.659E+9	0.023	----	6.5871	SOLVENT PEAK	----	< min rt	
1.0647	1011	0.018	----	8.6939		----	< min rt	
1.1918	596	0.013	----	9.5488		----		
1.2542	756	0.012	----	9.9676		----		
1.2828	403	0.010	----	10.1270		----		
1.3869	962	0.013	----	10.6774		----		
1.4050	466	0.010	----	10.7735		----		
1.4334	1176	0.009	1.067	10.9233	Sum In Feature 2	0.2	ECL deviates -0.007	12:0 aldehyde ?
1.5753	360	0.009	----	11.5527		----		
1.6112	834	0.011	1.042	11.7094	12:0 anteiso	0.1	ECL deviates -0.001	Reference -0.004
1.6720	7674	0.009	----	11.9752		----		
1.7958	590	0.013	----	12.4499		----		
1.8360	405	0.011	----	12.6026		----		
1.8656	966	0.009	1.015	12.7151	13:0 anteiso	0.	ECL deviates 0.001	Reference -0.005
1.9244	2469	0.010	----	12.9386		----		
2.0783	741	0.010	0.999	13.4767	12:0 3OH	0.1	ECL deviates -0.006	
2.1174	466	0.008	----	13.6116		----		
2.1480	876	0.010	0.995	13.7174	14:0 anteiso	0.1	ECL deviates -0.001	Reference -0.010
2.1685	387	0.010	----	13.7881		----		
2.2249	122121	0.009	0.990	13.9831	14:0	19.6	ECL deviates -0.017	
2.3703	2840	0.008	0.983	14.4550	15:1 iso G	0.	ECL deviates -0.005	
2.4183	980	0.009	----	14.6119		----		
2.4486	800	0.009	0.980	14.7106	15:0 anteiso	0.1	ECL deviates -0.014	
2.4975	1439	0.011	0.978	14.8702	15:1 w6c	0.2	ECL deviates -0.005	
2.5237	39781	0.009	----	14.9559		----		
2.6101	910	0.011	0.973	15.2312	14:0 2OH	0.1	ECL deviates -0.002	
2.6953	5066	0.009	0.970	15.5016	Sum In Feature 2	0.8	ECL deviates 0.001	16:1 iso I/14:0 3OH
2.7125	1764	0.009	----	15.5561		----		
2.7276	844	0.010	----	15.6040		----		
2.7619	449	0.010	----	15.7128		----		
2.7958	2267	0.013	----	15.8204		----		
2.8468	305636	0.009	0.965	15.9821	16:0	47.8	ECL deviates -0.018	
2.8683	406	0.013	----	16.0503		----		
2.9215	540	0.012	----	16.2189		----		
2.9945	190575	0.009	0.961	16.4500	Sum In Feature 9	29.	ECL deviates 0.003	17:1 iso w9c
3.0199	786	0.013	0.960	16.5301	15:0 3OH	0.1	ECL deviates -0.003	
3.0761	429	0.010	----	16.7080		----		
3.1254	771	0.015	----	16.8642		----		
3.1621	9721	0.009	----	16.9804		----		
3.2457	766	0.013	----	17.2469		----		
3.3070	789	0.009	----	17.4423		----		
3.3353	4152	0.010	----	17.5325		----		
3.3508	1657	0.010	----	17.5821		----		
3.3997	1686	0.009	0.950	17.7380	Sum In Feature 5	0.	ECL deviates 0.007	18:0 ante/18:2 w6,9c
3.4112	3120	0.010	----	17.7747		----		
3.4261	1384	0.010	----	17.8221		----		
3.4751	18806	0.009	----	17.9787		----		
3.6160	32522	0.009	----	18.4376		----		
3.9583	809	0.011	----	19.5667		----		
4.0825	419	0.010	----	19.9811		----		
4.2310	1030	0.011	----	20.4769		----	> max rt	
----	6243	---	----	----	Summed Feature 2	1.0	12:0 aldehyde ?	unknown 10.9525
----	-----	---	----	----		----	16:1 iso I/14:0 3OH	14:0 3OH/16:1 iso I
----	1686	---	----	----	Summed Feature 5	0.	18:0 ante/18:2 w6,9c	18:2 w6,9c/18:0 ante
----	190575	---	----	----	Summed Feature 9	29.	16:0 10-methyl	17:1 iso w9c

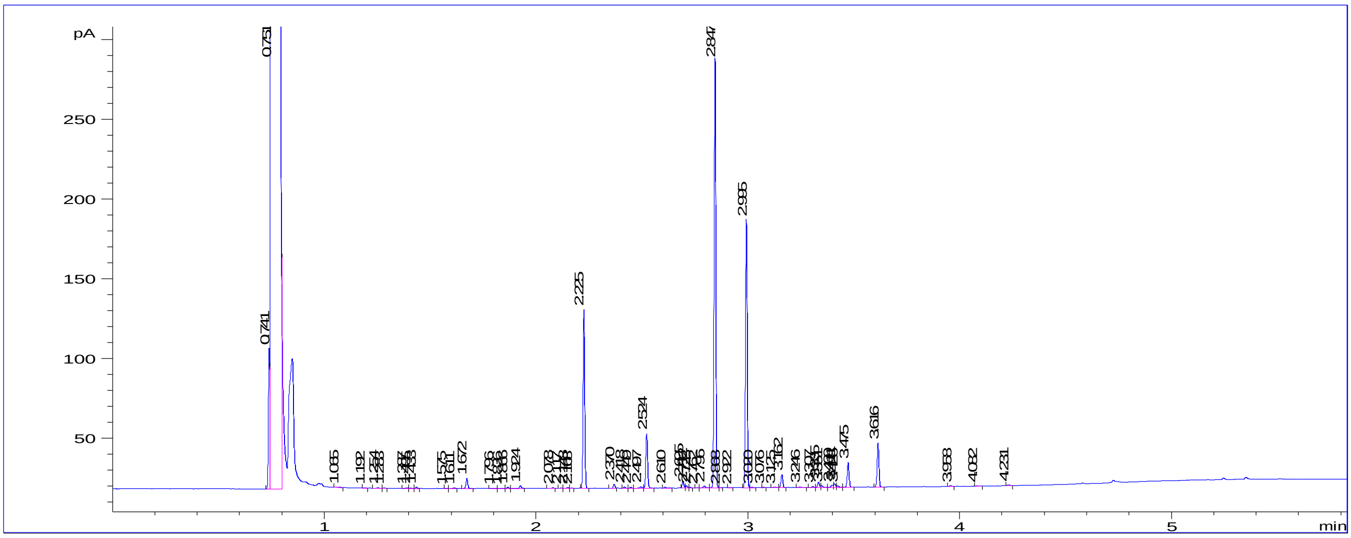
pA
250
200
150
100
50
1
2
3
4
5
min

RT	Response	Ar/Ht	RFact	ECL	Peak Name	Percent	Comment1	Comment2
0.7404	34842	0.006	----	6.5456		----	< min rt	
0.7500	1.748E+9	0.022	----	6.6103	SOLVENT PEAK	----	< min rt	
1.2529	1041	0.011	1.101	9.9988	10:0	0.1	ECL deviates -0.001	Reference 0.002
1.3910	531	0.012	----	10.7316		----		
1.5736	675	0.014	----	11.5765		----		
1.6099	622	0.010	----	11.7354		----		
1.6292	638	0.009	----	11.8198	unknown 11.825	----	ECL deviates -0.005	
1.6703	16586	0.009	1.032	11.9994	12:0		ECL deviates -0.001	Reference 0.001
1.8352	700	0.009	1.016	12.6254	13:0 iso	0.	ECL deviates 0.002	Reference 0.003
1.8642	918	0.010	----	12.7355		----		
1.9234	1913	0.011	1.009	12.9600	13:1 at 12-13	0.2	ECL deviates 0.002	
2.1161	852	0.011	0.997	13.6285	14:0 iso	0.1	ECL deviates 0.001	Reference 0.000
2.1467	770	0.009	----	13.7343		----		
2.1757	46788	0.009	----	13.8343		----		
2.1998	2766	0.010	0.992	13.9173	14:1 w5c	0.3	ECL deviates 0.001	
2.2237	225209	0.008	0.990	14.0001	14:0	25.6	ECL deviates 0.000	Reference 0.000
2.3689	2710	0.010	0.984	14.4742	Sum In Feature 1	0.3	ECL deviates -0.002	15:1 iso H/13:0 3OH
2.3842	621	0.010	----	14.5241		----		
2.4172	2073	0.009	0.981	14.6317	15:0 iso	0.2	ECL deviates 0.000	Reference -0.001
2.4464	1512	0.009	0.980	14.7273	15:0 anteiso	0.	ECL deviates 0.002	Reference 0.001
2.4748	442	0.009	0.979	14.8200	15:1 w8c	0.	ECL deviates 0.006	
2.4932	1032	0.012	0.978	14.8799	15:1 w6c	0.1	ECL deviates 0.005	
2.5226	15331	0.010	----	14.9760	unknown 14.969	----	ECL deviates 0.007	
2.6811	486	0.010	0.972	15.4802	16:1 iso H	0.	ECL deviates -0.002	
2.6950	751	0.009	0.971	15.5243	Sum In Feature 2	0.	ECL deviates 0.009	14:0 3OH/16:1 iso I
2.7284	878	0.011	0.970	15.6304	16:0 iso	0.1	ECL deviates -0.003	Reference -0.004
2.7594	479	0.011	0.969	15.7291	16:0 anteiso	0.	ECL deviates 0.002	
2.7945	177780	0.009	0.968	15.8405	Sum In Feature 3	19.	ECL deviates 0.000	16:1 w7c/16:1 w6c
2.8218	12188	0.010	0.967	15.9272	16:1 w5c	1.	ECL deviates -0.001	
2.8458	367590	0.009	0.967	16.0034	16:0	40.8	ECL deviates 0.003	Reference 0.002
2.8996	557	0.013	0.965	16.1737	15:0 iso 3OH	0.	ECL deviates 0.012	
2.9209	527	0.011	----	16.2409		----		
2.9403	529	0.010	----	16.3025		----		
2.9577	322	0.008	----	16.3576		----		
2.9705	650	0.010	----	16.3979		----		
2.9931	63440	0.009	----	16.4695		----		
3.0147	1151	0.012	----	16.5380		----		
3.0455	626	0.012	0.961	16.6355	17:0 iso	0.	ECL deviates -0.002	Reference -0.003
3.0750	616	0.011	0.960	16.7288	17:0 anteiso	0.	ECL deviates -0.004	Reference -0.006
3.1136	996	0.013	----	16.8510		----		
3.1233	439	0.008	0.959	16.8817	17:1 w6c	0.	ECL deviates 0.001	
3.1611	3895	0.009	0.958	17.0014	17:0	0.4	ECL deviates 0.001	Reference 0.000
3.2151	797	0.010	0.957	17.1734	16:0 iso 3OH	0.	ECL deviates -0.001	
3.2790	586	0.009	----	17.3775		----		
3.3343	2873	0.010	0.954	17.5537	16:0 3OH	0.3	ECL deviates 0.006	
3.3499	723	0.010	0.953	17.6035	18:3 w6c (6,9,12)	0.	ECL deviates 0.003	
3.3981	3344	0.008	0.952	17.7572	Sum In Feature 5	0.	ECL deviates 0.001	18:2 w6,9c/18:0 ante
3.4096	8418	0.009	0.952	17.7940	18:1 w9c	0.9	ECL deviates 0.000	
3.4256	21495	0.010	0.951	17.8450	Sum In Feature 8	2.	ECL deviates -0.002	18:1 w7c
3.4549	1376	0.010	0.951	17.9385	18:1 w5c	0.	ECL deviates 0.001	
3.4738	26352	0.009	0.950	17.9986	18:0	2.	ECL deviates -0.001	Reference -0.003
3.5353	911	0.012	0.949	18.1995	17:0 iso 3OH	0.1	ECL deviates 0.006	
3.5664	2660	0.010	0.948	18.3009	17:0 2OH	0.	ECL deviates 0.013	
3.6151	10087	0.010	----	18.4597		----		
3.6604	587	0.013	----	18.6076		----		
3.6751	1091	0.021	----	18.6557		----		
3.9562	1032	0.013	0.936	19.5851	18:0 3OH	0.1	ECL deviates 0.005	
4.0812	735	0.012	0.931	20.0017	20:0	0.	ECL deviates 0.002	Reference 0.001
4.2293	912	0.012	----	20.4952		----	> max rt	
----	2710	---	----	----	Summed Feature 1	0.3	15:1 iso H/13:0 3OH	13:0 3OH/15:1 iso H
----	751	---	----	----	Summed Feature 2	0.	12:0 aldehyde ?	unknown 10.9525
----	-----	---	----	----		----	16:1 iso I/14:0 3OH	14:0 3OH/16:1 iso I
----	177780	---	----	----	Summed Feature 3	19.	16:1 w7c/16:1 w6c	16:1 w6c/16:1 w7c
----	3344	---	----	----	Summed Feature 5	0.	18:0 ante/18:2 w6,9c	18:2 w6,9c/18:0 ante
----	21495	---	----	----	Summed Feature 8	2.4	18:1 w7c	18:1 w6c

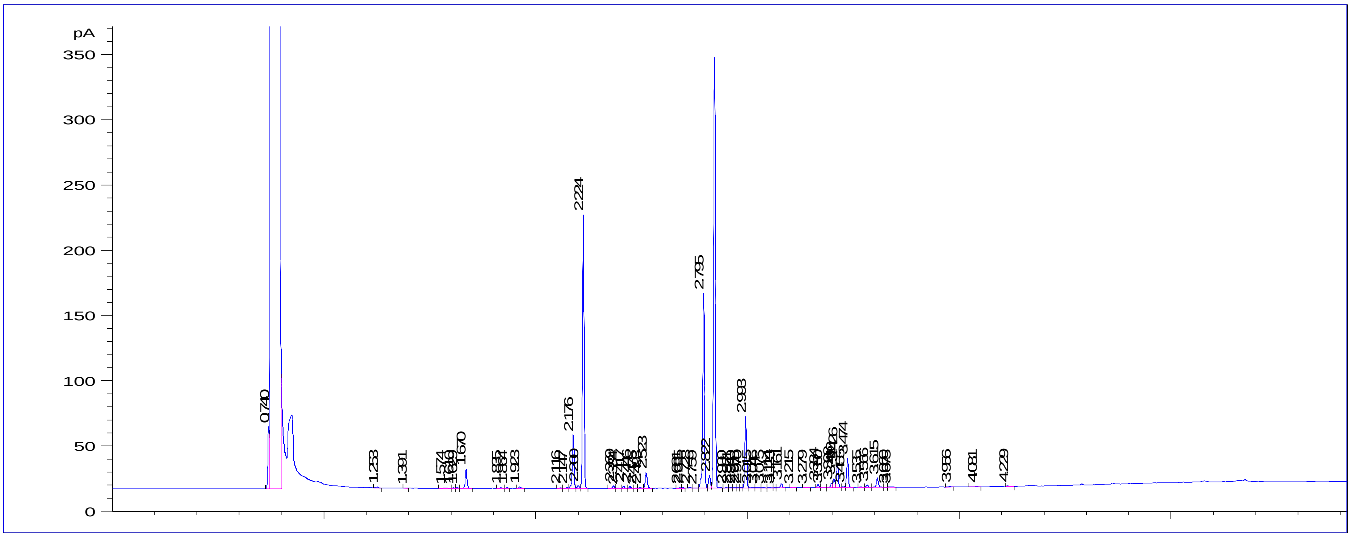
pA
350
300
250
200
150
100
50
0

Table S2a. Conserved Proteins (POCP) values between P01024^{T}, P01025^{T} and closely related type spec

Compared strain A	Compared strain B	POCP (%)
P01024^{T}	*Flavonifractor plautii* ATCC 29863^{T}	59.84
P01024^{T}	*Lawsonibacter celer* NSJ 47^{T}	55.84
P01024^{T}	*Pseudoflavonifractor capillosus* ATCC 29799^{T}	51.02
P01024^{T}	*Muriventricola aceti* H5_61^{T}	42.81
P01024^{T}	P01025^{T}	49.02
P01025^{T}	*Flintibacter hominis* New-19^{T}	53.02
P01025^{T}	*Muriventricola aceti* H5_61^{T}	50.46
P01025^{T}	*Pseudoflavonifractor capillosus* ATCC 29799^{T}	48.18
